# Genomic Epidemiology and Global Population Structure of Exfoliative Toxin A-Producing *Staphylococcus aureus* Strains Associated With Staphylococcal Scalded Skin Syndrome

**DOI:** 10.3389/fmicb.2021.663831

**Published:** 2021-08-18

**Authors:** Taj Azarian, Eleonora Cella, Sarah L. Baines, Margot J. Shumaker, Carol Samel, Mohammad Jubair, David A. Pegues, Michael Z. David

**Affiliations:** ^1^Burnett School of Biomedical Sciences, University of Central Florida, Orlando, FL, United States; ^2^Department of Microbiology and Immunology, The University of Melbourne at The Peter Doherty Institute for Infection and Immunity, Melbourne, VIC, Australia; ^3^Division of Infectious Diseases, University of Pennsylvania, Philadelphia, PA, United States; ^4^Department of Healthcare Epidemiology, Infection Prevention and Control, University of Pennsylvania, Philadelphia, PA, United States

**Keywords:** ETA, temperate bacteriophage, *Staphylococcus aureus*, exfoliative toxin A, phylogenenetic analysis, bacteriophage (phage), staphylococcal scalded skin syndrome, genomic epidemiology

## Abstract

Staphylococci producing exfoliative toxins are the causative agents of staphylococcal scalded skin syndrome (SSSS). Exfoliative toxin A (ETA) is encoded by *eta*, which is harbored on a temperate bacteriophage ΦETA. A recent increase in the incidence of SSSS in North America has been observed; yet it is largely unknown whether this is the result of host range expansion of ΦETA or migration and emergence of established lineages. Here, we detail an outbreak investigation of SSSS in a neonatal intensive care unit, for which we applied whole-genome sequencing (WGS) and phylogenetic analysis of *Staphylococcus aureus* isolates collected from cases and screening of healthcare workers. We identified the causative strain as a methicillin-susceptible *S. aureus* (MSSA) sequence type 582 (ST582) possessing ΦETA. To then elucidate the global distribution of ΦETA among staphylococci, we used a recently developed tool to query extant bacterial WGS data for biosamples containing *eta*, which yielded 436 genomes collected between 1994 and 2019 from 32 countries. Applying population genomic analysis, we resolved the global distribution of *S. aureus* with lysogenized ΦETA and assessed antibiotic resistance determinants as well as the diversity of ΦETA. The population is highly structured with eight dominant sequence clusters (SCs) that generally aligned with *S. aureus* ST clonal complexes. The most prevalent STs included ST109 (24.3%), ST15 (13.1%), ST121 (10.1%), and ST582 (7.1%). Among strains with available data, there was an even distribution of isolates from carriage and disease. Only the SC containing ST121 had significantly more isolates collected from disease (69%, *n* = 46) than carriage (31%, *n* = 21). Further, we identified 10.6% (46/436) of strains as methicillin-resistant *S. aureus* (MRSA) based on the presence of *mecA* and the SCC*mec* element. Assessment of ΦETA diversity based on nucleotide identity revealed 27 phylogroups, and prophage gene content further resolved 62 clusters. ΦETA was relatively stable within lineages, yet prophage variation is geographically structured. This suggests that the reported increase in incidence is associated with migration and expansion of existing lineages, not the movement of ΦETA to new genomic backgrounds. This revised global view reveals that ΦETA is diverse and is widely distributed on multiple genomic backgrounds whose distribution varies geographically.

## Introduction

Outbreak investigations for bacterial pathogens in the healthcare setting have been transformed in recent years with the advent of pathogen whole-genome sequencing (WGS). Several investigations of *Staphylococcus aureus* outbreaks in neonatal intensive care units (NICUs) have been reported (Köser et al., 2012; Nübel et al., 2013; Azarian et al., 2015, 2016). By interrogating the genomes of emerging pathogens, researchers are able to assess their clonal distribution (i.e., population structure) and migration as well as the genomic determinants of virulence and antimicrobial resistance. Since the first genomes of *S. aureus* were published in 2001 (Kuroda et al., 2001), studies have described the evolution and dissemination of epidemiologically important strains (Kuroda et al., 2001; Gonzalez et al., 2006). The application of genomics was subsequently expanded to track significant changes in the epidemiology of virulent lineages in hospital outbreaks and on larger geographic and temporal scales (Harris et al., 2010; Alam et al., 2015).

Genomic studies of *S. aureus* have historically focused on methicillin-resistant *S. aureus* (MRSA) due to challenges in treatment and poorer clinical outcomes (Dasenbrook et al., 2010; Harkins et al., 2018; Kanjilal et al., 2018). Yet methicillin-susceptible *S. aureus* (MSSA) strains continue to cause significant morbidity and mortality (Jackson et al., 2019). In particular, some *S. aureus* strains produce epidermolytic staphylococcal exfoliative toxins (ETs), extracellular proteins that cause separation of the epidermal layer of the skin. These strains cause a serious but rare condition known as staphylococcal scalded skin syndrome (SSSS), predominantly in children (Ladhani et al., 1999). Two ETs have been identified among staphylococci: ETA, encoded by the chromosomally located gene *eta*, and ETB, encoded by gene *etb* that is found on a large plasmid (Yamaguchi et al., 2000). ETA is harbored by a temperate bacteriophage, ΦETA, that is able to lysogenize susceptible strains (Yamaguchi et al., 2000).

The first clinical descriptions of SSSS were in the mid-19th century (Ladhani et al., 1999), and due to its distinctive presentation, pediatric outbreaks are frequently reported (Anthony et al., 1972; Florman and Holzman, 1980; El Helali et al., 2005). Molecular data, on the other hand, are limited (Murono et al., 1988; Yamaguchi et al., 2002; Doudoulakakis et al., 2017). As the genes coding for ETA and ETB are found on mobile genetic elements (MGEs), their distribution in the *S. aureus* population should seemingly be diverse. Yet ET-producing strains are often restricted to a few clonal complexes (CCs) and are principally oxacillin susceptible (Braunstein et al., 2014). Recently, an increased incidence of SSSS in the United States has been reported (Staiman et al., 2018; Hultén et al., 2019). A study by Hultén et al. (2019) reported an increase in cases of SSSS at a children’s hospital from 2.3/10,000 admissions in 2008 to 52.6/10,000 admissions in 2017. The majority of causative isolates identified were MSSA strains belonging to CC 121, a lineage previously associated with SSSS (Botka et al., 2017; Doudoulakakis et al., 2017). The genomic epidemiology of ET-positive strains, particularly regarding the distribution of ET among staphylococci, remains unexplored.

Here, we describe an outbreak investigation of MSSA-associated SSSS in a NICU. We identified the epidemic strain as sequence type (ST) 528 possessing *eta*. Using Illumina short-read and nanopore long-read sequencing data, we generated and published a high-quality reference genome for the outbreak clone (Cella et al., 2021). Using published WGS data in the European Nucleotide Archive (ENA), we identified all deposited *S. aureus* genomes possessing *eta*, which we then analyzed to determine the distribution of ΦETA as well as the diversity of ETA phages.

## Materials and Methods

### Outbreak Investigation and Healthcare Worker Screening

Ongoing surveillance for healthcare-associated infections identified a putative cluster of temporally related SSSS infections among neonates hospitalized in the NICU of a tertiary care medical center. Microbiological analysis identified the causative agent as MSSA susceptible to all tested antimicrobials. Due to the comparatively low incidence of SSSS, an epidemiological association was suspected, and an investigation was initiated. In addition to the implementation of environmental control measures, we carried out MSSA carriage screening of healthcare workers who had contact with the incident cases. Clinical isolates from case patients (MSSA_SSSS_01–MSSA_SSSS_04) and carriage isolates from healthcare workers underwent WGS and phylogenetic analysis.

### Whole-Genome Sequencing and Phylogenetic Analysis

gDNA extraction was carried out using Qiagen DNeasy blood and tissue kit (Qiagen, Germantown, MD, United States) according to the manufacturer’s instruction; and gDNA quantification was carried out using a Qubit. WGS short-read libraries were constructed using the Illumina Nextera Flex library prep kit, which were subsequently sequenced on an Illumina HiSeq using 250 cycle V3 chemistry flow cell to produce 2 × 250 paired-end reads with a target idealized coverage of 50×. After sequencing, per sample idealized coverage was calculated, and quality was assessed using FastQC. Raw reads were quality filtered with Trimmomatic v.0.39 using the following settings: SLIDINGWINDOW:10:20 MINLEN:31 TRAILING:20 (Chen et al., 2014). Multilocus sequence typing (MLST) was inferred using srst2 using Illumina data based on the *S. aureus* MLST database^1^ (Inouye et al., 2014). *De novo* genome assembly of WGS data was performed using Unicycler v0.4.8 (Wick et al., 2017) with default options and annotated with Prokka v1.14.6 using the MSSA_SSSS_01 (NZ_CP061349.1) reference genome annotation to preferentially annotate coding sequences (Seemann, 2014). For a single isolate collected from the index case (MSSA_SSSS_01), we generated a high-quality complete genome using long-read data from the Oxford Nanopore MinION. The methods for assembly, polishing, and annotation of MSSA_SSSS_01 are described in detail elsewhere (Cella et al., 2021). Pangenome analysis was performed using Roary v.3.12 (Page et al., 2015), and single-nucleotide polymorphism (SNP) alignment was extracted from the core genome (i.e., loci present in >99% of the sample) using snp-sites v2.4.0.^2^ A maximum likelihood (ML) phylogeny was inferred with IQTREE v1.6.8 using the ASC_GTRGAMMA substitution model with 100 bootstrap replicates (Nguyen et al., 2015). For isolates phylogenetically clustering with the SSSS cases, WGS data were mapped to MSSA_SSSS_01 using Snippy v4.6.0,^3^ and a SNP alignment was used to infer an ML phylogeny as described above. To infer transmission, the pairwise distances among highly related strains were assessed, and the ML phylogeny was interrogated.

### Identification of ETA + Global *Staphylococcus aureus* Strains and Investigation of Population Structure

To investigate the global distribution of *eta*, we used Bitsliced Genomic Signature Index (BIGSI) to query the entire global ENA repository of bacterial WGS datasets for biosamples containing *eta* (NCBI Reference Sequence: NP_510960.1) (Yamaguchi et al., 2000; Bradley et al., 2019). Biosamples with BLAST identity over 90% were downloaded, assembled, and annotated as described above. All associated metadata were downloaded from public WGS data repository. To determine the year and country of isolation and source (carriage or disease), we conducted an extensive review of the literature using all available accession numbers, sample names, and aliases. Metadata tables were obtained from Supplementary Material of published studies. If published metadata were not available, study authors were contacted. Samples were deduplicated based on metadata (e.g., only one isolate was selected if multiple isolates were collected from a single study participant). In addition, samples were excluded if we could not verify the presence of *eta* in the *de novo* assembly and if the assembly failed quality check (contig >331, assembly length >3,100,000 or <2,650,000). For all remaining isolates, we performed pangenome and ML phylogenetic analysis and assigned MLST as described above. SNPs in the core genome were then used to assess population structure with fastBAPS (Tonkin-Hill et al., 2019). Based on the population structure and MLST analyses, dominant lineages were identified. For these lineages, we repeated the pangenome analysis and inferred a core genome phylogeny as described above. Abricate with default settings was used to detect the genotypic antibiotic resistance determinants.^4^ For MRSA isolates, staphylococcal cassette chromosome *mec* (SCC*mec*) type was determined by assessing ccr and mec gene complexes from assemblies using SCC*mec* finder tool^5^ (International Working Group on the Classification of Staphylococcal Cassette Chromosome Elements (IWG-SCC), 2009). MLST, SCC*mec*, and antibiotic resistance results were mapped on the core genome phylogeny using *ggTree* implemented in Rstudio running R v 3.6.0.

### Dating of the Outbreak Lineage

Global strains belonging to the same lineage as the outbreak strain (represented by MSSA_SSSS_01, *n* = 30) were dated using Bayesian coalescent analysis. First, WGS data were mapped to MSSA_SSSS_01, using Snippy v4.6.0 as described above, to produce a reference-based alignment. Providing the alignment to Gubbins v2.4.1, we identified SNPs introduced through recombination, which were then censored as they interfere with phylogenetic inference. An ML phylogeny was subsequently inferred from the recombination-censored alignment (Croucher et al., 2014). The ML phylogeny and collection year of each sample were used as input for BactDating v1.0, a tool used to perform Bayesian dating of a bacterial phylogenetic tree (Didelot et al., 2018). We assessed temporal signal (i.e., evidence of clock-like evolution), using the *roottotip* function, which regresses the year of collection to the tree distance. BactDating was then run with a Markov chain Monte Carlo (MCMC) chain length of 2,000,000 to estimate the molecular clock and rate of coalescence. The resulting evolutionary rate was assessed, and the time-scaled tree was visualized.

### ΦETA Diversity and Population Structure

We then sought to investigate the population structure of the *eta*-containing phages found in our dataset. Phage-containing contigs were searched using *eta* and *int* genes from the *eta* phage in MSSA_SSSS_01 as targets (NZ_CP061349.1, locus tags IC588_RS04295 and IC588_RS03960), aligned based on the target sequences, annotated using contig-puller v2.0,^6^ and trimmed in Geneious Prime^®^ 2020.1.2 (Biomatters Ltd., Auckland, New Zealand) at the attachment site sequences, reported in Yamaguchi et al. (2000). Genome assemblies in which both attachment sites were identified and recovered on a single contig were designated complete, and these sequences were analyzed using Phaster to determine the closest published phage genome (Arndt et al., 2016). Synteny of the phage integration sites was visually inspected, and *int* gene homology was assessed using fastablasta v0.5^7^ with a 95% nucleotide identity threshold to the MSSA_SSSS_01 *int* gene as a reference (NZ_CP061349.1, locustag IC588_RS03960). To recover putative phage sequences for the remaining genome assemblies with fragmented assemblies, we performed ortholog clustering of phage genes. The gene presence/absence output from Roary pangenome analysis (described above, but re-run including all genome assemblies and the complete ΦETA) was filtered with the goal of identifying gene clusters that were (i) unique to ΦETA, (ii) present as a single copy, and (iii) full length/complete (i.e., not pseudo-genes). This was accomplished by excluding any clusters that were not represented in the complete phage sequences, clusters with multi-copy or truncated genes, and clusters inconsistently identified in the whole genome vs. phage sequence (i.e., the cluster was only detected in the whole genome assembly but absent in the complete ΦETA sequence for an isolate; the gene cluster being phage associated, but not unique to ΦETA). The dataset was then filtered so that each isolate was only represented once. A presence/absence matrix was constructed with the 177 gene clusters remaining post-filtering, and a midpoint rooted UPGMA phylogenetic tree was inferred using SplitsTree4 v4.14.6.

To define groups and further resolve the diversity and population structure of ΦETA, complete phage genomes (*n* = 139) were aligned using Clustal Omega v1.2.4 (Sievers and Higgins, 2018) to estimate pairwise nucleotide distances. Phages were clustered based on a 95% nucleotide identity threshold as suggested in Adriaenssens and Brister (2017) and assigned “G00” numbers. Pairwise nucleotide distances were visualized using a heatmap. This clustering approach was used to determine a cutoff for grouping in the phage gene presence/absence matrix, to define phage population structure for all genomes. Phages were clustered if they shared the same presence/absence profile ± 2 gene clusters, and then assigned phylogroup “P00” numbers.

## Results

### Outbreak Investigation

**FIGURE 1:**
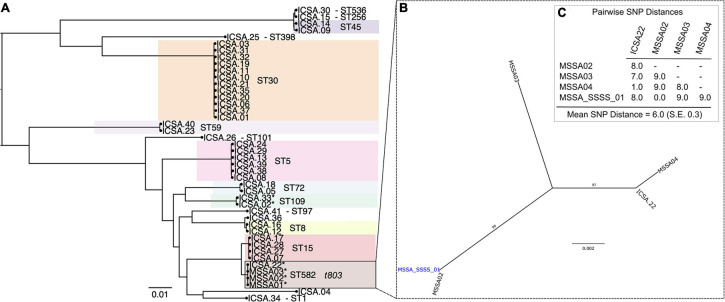
Phylogenetic analysis of methicillin-susceptible *Staphylococcus aureus* isolates from colonized healthcare workers (ICSA strains) and clinical cases of staphylococcal scalded skin syndrome (SSSS) (MSSA01–MSSA04). **(A)** Maximum likelihood (ML) phylogeny of 41 carriage isolates inferred from a core genome alignment of 1,989 genes totaling 1,855,652 and containing 80,495 single-nucleotide polymorphisms (SNP), indicating multilocus sequence type (ST). **(B)** ML phylogeny of four clinical SSSS isolates and a single related healthcare worker carriage isolate. Whole-genome sequencing data were mapped to the closed MSSA_SSSS_01 reference genome. **(C)** Pairwise SNP distances of isolates included in the phylogeny shown in panel **(B)**.

Analysis of MSSA isolates from four patients hospitalized with SSSS identified that all belonged to ST582 (CC15) and possessed the ΦETA prophage that harbors *eta*. Index strain MSSA_SSSS_01 was fully resolved using hybrid genome assembly approach, and expression of *eta* was confirmed using qRT-PCR as described elsewhere (Cella et al., 2021). Assessment of pairwise genetic distances suggested that all were related by recent transmission events (Figure 1C), and epidemiological analysis of length of stay in the unit showed that two of the four patients had overlapping lengths of stay (Supplementary Figure 1). Nares screening of 89 healthcare workers who had contact with the case patients revealed 41 asymptomatic carriers of MSSA. Phylogenetic analysis showed a diverse population of carried MSSA strains dominated by ST30 and ST5 (Figure 1). Most significantly, isolates from SSSS cases clustered with an isolate obtained from a single healthcare worker who had contact with the SSSS case patients during their hospitalization. All five isolates, when considered together, were highly genetically related, suggesting that the healthcare worker is a part of the transmission chain. However, the directionality of transmission was difficult to resolve. As a result of this finding, the healthcare worker was decolonized using the following protocol: oral doxycycline (100 mg) and rifampin (600 mg) twice daily for 7 days, intranasal mupirocin (2% ointment) and chlorhexidine gluconate 0.12% oral rinse twice daily for 5 days, and daily chlorhexidine baths. Screening of multiple body sites performed 7 and 28 days after initiating the regimen confirmed decolonization. Enhanced environmental cleaning was also implemented in the unit. Subsequently, no additional SSSS cases were identified.

### Global Distribution of Strains Possessing ΦETA

**FIGURE 2:**
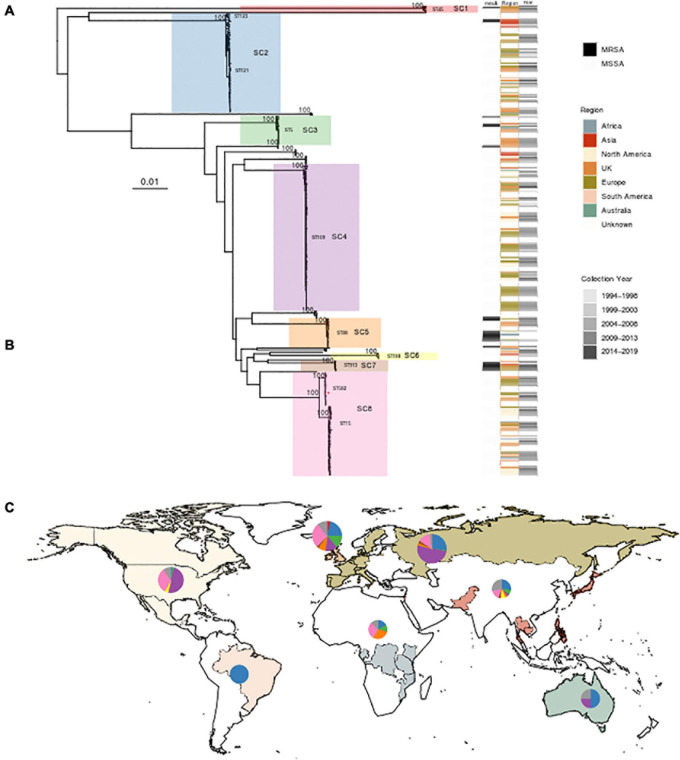
Phylogenetic analysis and global distribution of 436 *Staphylococcus aureus* isolates that possess the gene encoding exfoliative toxin A (*eta*). **(A)** Maximum likelihood (ML) phylogeny of 436 isolates, including three strains from this study, inferred from a core genome alignment of 1,794 genes totaling 1,615,001 nucleotides in length and containing 63,517 single-nucleotide polymorphisms. Bootstrap values showing statistical support for tree topology are annotated on the branches. Lineages [sequence clusters (SCs)] as identified through statistical analysis of population structure are highlighted on the phylogeny. A red star on SC8 indicates the location of the ST582 outbreak isolate MSSA_SSSS_01. The SC and multilocus sequence types (MLST) are shown next to lineages. Strains possessing *mecA*, geographic region of isolation, and year of collection are indicated on the heatmap to the right of the phylogeny. **(B)** Table showing the distribution of 436 strains among SCs with the color corresponding to the heatmap in panel A. The MLST types comprising each SC are also listed. **(C)** World map illustrating sampled countries and regions with respective proportion of isolates belonging to each SC. Countries are colored according to region membership, and the region colors correspond to the heatmap in panel **(A)** and table in panel **(B)**. The proportion of isolates belonging to major SC is indicated by the pie chart. The United Kingdom is presented separately from the rest of Europe due to the number of sampled strains and the difference in population structure. The size of the pie chart is scaled by the proportion of the total number of strains sampled from each region. Mapping was performed using R package maps v3.3.0.

Querying extant WGS data identified 434 genomes whose *de novo* assembly contained *eta* and passed quality parameters; two additional ST582 samples identified in the outbreak investigation detailed above were included. After review and abstraction of all associated metadata, 290 genomes were complete, having information on year and country of collection as well as source (Supplementary File 1). The sample spanned collection between 1994 and 2019 from 32 countries, classified into seven regions. The greatest proportion of the sample was collected from the United Kingdom (*n* = 126), the United States (*n* = 79), and Belgium (*n* = 55) (Figure 2). Analysis of population structure identified eight dominant lineages [sequence clusters (SCs)] accounting for 91.7% of the overall population. The most prevalent MLST types included ST109 (24.3%), ST15 (13.1%), ST121 (10.1%), and ST582 (7.1%). ST15 and ST582 belong to the same CC (CC15); together, they accounted for 20.2% of the total population (Figure 2). Among 297 strains with available data, there was almost an equal distribution of isolates from asymptomatic carriage (47%) and disease (53%). Only SC2, which contains ST121, appeared to have more isolates collected from disease (69%, *n* = 46) than carriage (31%, *n* = 21). Phylogenetic analysis of each SC showed clear spatial structure even within STs (Supplementary Figures 2, 3). For example, ST121 belonging to SC2 comprised two clades, one prevalent in the United Kingdom and the other in Central Europe. ST109 of SC4 was largely limited to North America and Central Europe. ST15 of SC8 was the most widely distributed, while ST582, comprising our outbreak strain, of the same SC, appeared to have only recently emerged in North America. Finally, we identified 10.6% (46/436) of strains as MRSA based on the presence of *mecA* and the SCC*mec* element. MRSA strains were most often ST88 (18/46), ST913 (9/46), and ST8 (5/46), with SCC*mec* type IVa (2B) the most common among all MRSA strains. Of 46 *eta* carrying MRSA strains, 20 were from undefined disease, six were from carriage, and the remainder had no source information available. Further, while several studies confirmed oxacillin resistance using phenotypic testing, none specified whether ET production or *eta* expression was assessed. Among other genotypic resistance determinants in the 436 *S. aureus* isolates, the macrolide resistance determinant *erm* was the most common; 163 isolates possessed *ermA* (*n* = 120), *ermB* (*n* = 1), or *ermC* (*n* = 42) followed by tetracycline efflux pump *tetK* (*n* = 31) or *tetM* (*n* = 3).

### Dating of ST582

Because the isolates identified during our outbreak investigation belonged to ST582, a relatively infrequent ST among clinical *S. aureus* isolates, we sought to further elucidate the emergence of the lineage, which is most closely related to ST15–CC15, designated SC8 in our analysis of population structure (Figure 2). Analysis of recombination identified that the average number of polymorphisms introduced by recombination compared with mutation (*r/m*) was 6.7. Root-to-tip analysis showed significant temporal signal, allowing for coalescent analysis (Supplementary Figure 4). Dating of this lineage identified an evolution rate of 4.39 [95% highest posterior density (HPD) 1.28–9.46] SNPs/genome/year and a date of the most recent common ancestor of 1931.8 (95% HPD 1785.1–1990.8), which places the split of ST15 and ST582 prior to that date.

### ΦETA Diversity and Population Structure

**FIGURE 3:**
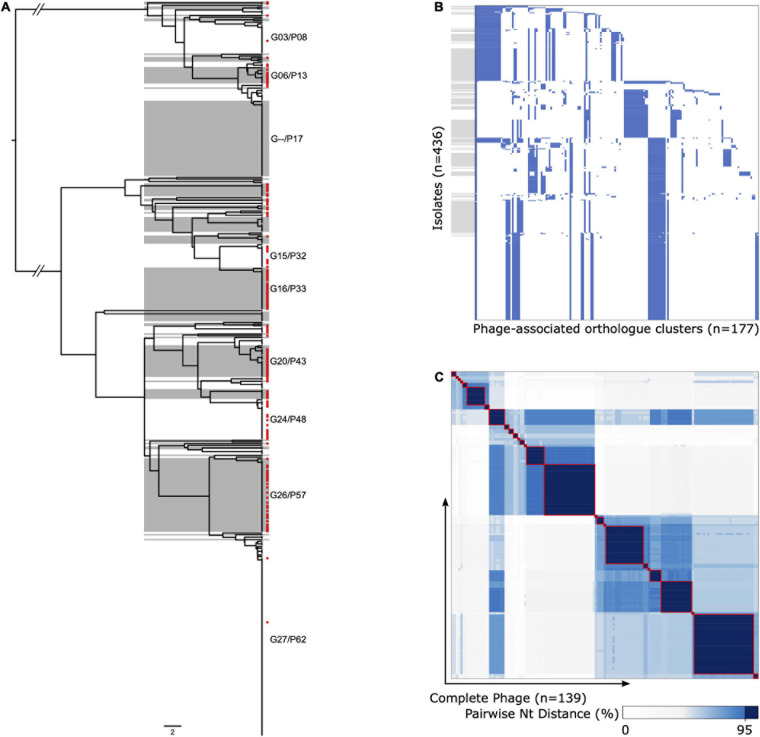
Diversity and population structure of ΦETA. **(A)** Phage gene tree (scale = 2/177 genes) illustrating the clustering of ΦETA. Alternating gray/white shading indicates phage phylogroups based on the presence/absence of gene ortholog clusters, guided by the nucleotide identity of complete prophage sequences as described in the *Methods*. Red tip symbols indicate isolates with complete prophage sequences. Phylogroups with ≥10 representative are annotated on the tree. **(B)** Gene presence/absence matrix for all isolates used to generate gene tree (blue, present; white, absent). The *y*-axis is ordered by the phage tree order shown in panel **(A)**, and gray boxes align with phage phylogroups. The *x*-axis is ordered based on gene presence descending across tree. **(C)** Heatmap of pairwise nucleotide distances between the complete phage sequences [*n* = 139, red tip labels in panel **(A)**]. The gradient indicates percent nucleotide identity, with a consistent dark blue and red outline to highlight cluster with ≥95%.

We were able to extract 139 complete ΦETA sequences from 436 genome assemblies. Visual inspection of the integration sites identified high conservation of *int* gene (all sharing >98% nucleotide homology to MSSA_SSSS_01 *int* gene), the attL and attR sequences, and integration location. This suggests a conserved integrase type, and although variable in gene content, it is a conserved integration location. Assessment of ΦETA diversity based on nucleotide identity of complete phage sequences identified 27 groups designated as G01–G27, and gene content from the presence/absence matrix further resolved the structure into 62 phylogenetically congruent clusters, designated phylogroups as P01–P62 (Figure 3). While we were able to resolve phages into well-defined clusters, appreciable diversity was observed in both gene content (Figure 3B) and nucleotide identity (Figure 3C). In comparison with the population structure of the strains harboring them, ΦETA variants were relatively stable within lineages (Supplementary Figure 5). However, variation in phage phylogroup and cluster within lineages suggests repeated infection of susceptible strains by diverse ETA phages as well as the impact of recombination to phage gene content (Supplementary Figures 2, 3). Characterization of lineages, phage population structure, and published ETA phage genomes elucidated varied evolutionary history in phage acquisition even among closely related clades (Supplementary Table 1). Notably, ST121 strains belonging to SC2 all possessed a highly related (same phage cluster) ΦETA matching phiETA3 (NC_008799), while the closely related ST123 showed greater diversity in phage variation. SC4, largely composed of ST109 and closely related MLSTs, largely possessed a B166-like ΦETA. SC8, which contained our outbreak isolate, was the most segregated with ST15 isolates possessing ΦETA phages matching phiETA3 and ST582 strains B166. Lastly, MRSA strains belonging to ST913 (SC7), which was limited to Western Europe, possessed a novel ETA phage related to SA97 (NC_029010) that was not found in any other lineage (Figure 2A).

## Discussion

Staphylococcal scalded skin syndrome is one of the most well-defined *S. aureus* clinical syndromes in that it is clearly linked to a known genotype—the presence of genes encoding ETs. A recent increase in SSSS incidence has been reported from North America and abroad, suggesting either the recent emergence of lineages carrying ETs or an increase in horizontal transmission of the MGE-harboring genes encoding ETs. Applying genomic epidemiology to an outbreak investigation in a NICU, we identified the etiologic strain as an ETA + MSSA belonging to ST582, which is less commonly reported in the literature than ST121 as a cause of SSSS (Conceição et al., 2011; Rao et al., 2015; Hultén et al., 2019). We investigated the global distribution of ETA + *S. aureus* strains, finding both the diversity of lineages carrying ETA and the diversity of ETA temperate bacteriophages (referred to here as ΦETA) themselves to be significantly greater than previously appreciated. Furthermore, we dispel the notion that ΦETA is limited to MSSA by identifying at least three lineages that are composed largely of SCC*mec* + and *mecA* + strains with lysogenized ΦETA. These findings revise our understanding of the population structure of ETA + *S. aureus* and present a novel method of tracking genotypes of interest.

Genomic epidemiology is increasingly being applied to outbreak investigation in healthcare settings, and the recent increase in accessibility of sequencing technology and bioinformatics expertise has improved the utility of these data for real-time investigation. Here, temporally related SSSS cases in the NICU initiated an investigation, which involved screening healthcare workers caring for hospitalized infants. MSSA carriage among healthcare workers was in line with estimates from the general population. Most significantly, screening identified a colonized worker with a highly genetically related strain, indicative of recent transmission. While we cannot confirm the directionality of transmission (i.e., whether the healthcare worker was the source or acquired it from the incident outbreak case), the colonized healthcare worker explains the acquisition of patient 4 who had a non-overlapping stay with the other cases. This is further supported by the close genetic distance (1 SNP) separating the healthcare worker isolate and patient 4’s isolate. Decolonization of the healthcare worker as well as implementation of enhanced environmental cleaning resolved the outbreak, and no further cases to date have been identified.

A traditional approach in microbial population genomics is to identify a lineage of interest and track its emergence and spread. Often, these lineages possess an epidemiologically important phenotype such as increased virulence or antibiotic resistance conferred by genomic determinants carried on MGE such as ΦETA (Malachowa and Deleo, 2010). Here, instead, we reverse this approach by identifying the genetic element of interest, *eta*, and querying extant WGS data using a newly developed tool to elucidate the global distribution of *S. aureus* strains possessing ΦETA (Bradley et al., 2019). The majority of studies from which we obtained data were not focused on SSSS or related clinical syndromes (Nübel et al., 2013; Roisin et al., 2016; Moradigaravand et al., 2017; Mason et al., 2018); therefore, we believe this approach optimizes the use of published WGS data.

The temperate bacteriophages ΦETA, categorized as Sa1int phages, were previously thought to have low diversity and a relatively narrow host range, localized to few lineages, and limited to strains lacking SCC*mec* (Yamaguchi et al., 2000; Yoshizawa et al., 2000; Goerke et al., 2009). Further, while previous studies have shown that ΦETA is inducible and phage particles can convert susceptible strains (Yamaguchi et al., 2000; Yoshizawa et al., 2000), host specificity and the factors governing susceptibility remained obscure. Our revised global view reveals that ΦETA is diverse in terms of nucleotide identity and gene content and is widely distributed in multiple genomic backgrounds whose distribution varies geographically. The population is highly structured with eight dominant SCs that generally aligned with *S. aureus* CCs. ΦETA was observed to be relatively stable within lineages; however, it is apparent from the phage clustering analysis that recombination has generated considerable diversity within the prophage region and that these variations may be geographically specific. Interestingly, we observed relatively few singleton taxa divergent from main phylogenetic clades, suggesting that novel acquisitions of ΦETA are infrequent. Yet whether this results from the underlying variation in *S. aureus* population structure across geographies, low induction of ΦETA harboring strains, or limitations in host range requires further investigation.

Three SCs—SC4, SC8, and SC2—comprise greater than 74% of the total population of *eta* + strains. The most prevalent lineage, CC9 (SC4 in Figure 2), which includes ST9 and ST109 and has been previously associated with skin disorders and livestock-associated MRSA strains, represents nearly half of *eta* + isolates from North America and Europe (Růžičková et al., 2012; Botka et al., 2015). Following SC4, CC15 (SC8 in Figure 2), which includes ST582 and ST15, is the second most prevalent. This SC comprised two clades that possess considerable difference in their geographic distribution and phage content, with ST582 largely limited to Europe and North America. SC8 is globally distributed. ST15 and ST582 have been previously recognized to share a recent common ancestor, with ST582 possessing a 310-kb chromosomal replacement not found in ST15 (Didelot and Wilson, 2015). Furthermore, ST15 and ST582 each has a distinct ΦETA variant, suggesting separate acquisitions following their divergence, most likely at least 90 years ago based on dating of ST582. Finally, SC2, which comprised ST121 and ST123, is the third most prevalent lineage globally. While this lineage has previously had limited distribution in North America, recent reports suggest that it is now emerging there (Conceição et al., 2011; Hultén et al., 2019). SC2 is the only *eta* + lineage in our study for which our data suggest that it may be more commonly found in disease than carriage. This is supported by previous reports of ST121 as a hypervirulent lineage (Rao et al., 2015); however, an expanded sample of ETA– ST121 strains and additional analysis would be required to confirm these limited observations.

One promising finding is that antibiotic resistance is relatively limited among *eta* + *S. aureus* strains. The exception was the identification of sporadic clusters of MRSA, most often belonging to ST88, ST8, and ST913. MRSA strains possessing ΦETA were thought to be rare, as it was suggested that cross-immunity may inhibit co-carriage of SCC*mec* and ΦETA. Here, we show that it is more common than previously appreciated. The MRSA strains analyzed here were obtained from multiple studies, which is likely why their prominence was not appreciated until these data were aggregated. One lingering question is whether these strains produce ETA. From available metadata, we were able to determine that almost half of MRSA isolates were collected from cases of disease and were phenotypically resistant to oxacillin and β-lactam antibiotics. However, we are unable to confirm production of ETA nor infer production from clinical syndrome due to limited published information. Overall, the relationship between SCC*mec* and ΦETA requires further investigations. Further, relatively unknown lineages like ST913, which is found predominantly in the United Kingdom and Germany and possesses a novel ETA phage, will require further investigation and monitoring to determine the extent of its distribution and its epidemiological trajectories.

We have presented a comprehensive analysis of the diversity and distribution of ΦETA among *S. aureus*, an endeavor motivated by an outbreak investigation of SSSS and recent reports of increased incidence in North America. We illustrate the coevolution of ΦETA with its host *S. aureus*, which has resulted in considerable prophage variation that is geographically structured. Our finding that ΦETA is relatively stable within lineages suggests that the recent increase in incidence is associated with migration and clonal expansion of existing lineages, not the movement of ΦETA to new genomic backgrounds. Our study is not without limitations. Our sample and understanding of the distribution of ΦETA are reliant on the sampling of the included studies, which is biased by accessibility to WGS platforms and a disproportionate representation of antibiotic-resistant lineages of *S. aureus* in sequenced datasets. Further, as we are reliant on genomic data, we are unable to confirm phenotypic antibiotic susceptibility as well as the production of ETA, as it is known that some strains harboring ΦETA do not produce the toxin. Also, while we found that ΦETA is more widely distributed among *S. aureus* strains than previously understood, it is relatively limited in comparison with the extant population structure of *S. aureus*. Future studies should focus on understanding susceptibility of *S. aureus* to ΦETA lysogenization by comparing ETA- strains from the same STs. A full accounting of other MGE, including prophages and genomic elements, present in *eta* + strains would also help toward that goal.

## Data Availability Statement

The datasets presented in this study can be found in online repositories. The names of the repository/repositories and accession number(s) can be found in the article/Supplementary Material.

## Ethics Statement

The studies involving human participants were reviewed and approved by the University of Pennsylvania Institutional Review Board. Written informed consent from the participants’ legal guardian/next of kin was not required to participate in this study in accordance with the national legislation and the institutional requirements.

## Author Contributions

TA, MD, and DP contributed to conception and design of the study. MD, DP, MS, and CS performed data collection. MJ and EC contributed to whole genome sequencing data generation and analysis. TA wrote the first draft of the manuscript. SB and MD wrote sections of the manuscript. All authors contributed to the analysis and manuscript revision, read, and approved the submitted version.

## Conflict of Interest

The authors declare that the research was conducted in the absence of any commercial or financial relationships that could be construed as a potential conflict of interest.

## Publisher’s Note

All claims expressed in this article are solely those of the authors and do not necessarily represent those of their affiliated organizations, or those of the publisher, the editors and the reviewers. Any product that may be evaluated in this article, or claim that may be made by its manufacturer, is not guaranteed or endorsed by the publisher.

## Footnotes

1
http://saureus.mlst.net

2
https://github.com/sanger-pathogens/snp-sites

3
https://github.com/tseemann/snippy

4
https://github.com/tseemann/abricate

5
www.genomicepidemiology.org

6
https://github.com/kwongj/contig-puller

7
https://github.com/kwongj/fastablasta

## References

[B1] Adriaenssens E. Brister J. R. (2017). How to name and classify your phage: an informal guide. *Viruses* 9:70. 10.3390/v9040070 PMC5408676 28368359

[B2] Alam M. T. Read T. D. Petit R. A. Boyle-Vavra S. Miller L. G. Eells S. J. (2015). Transmission and microevolution of USA300 MRSA in U.S. households: evidence from whole-genome sequencing. *mBio* 6:e00054-15. 10.1128/mBio.00054-15 25759497 PMC4453535

[B3] Anthony B. F. Giuliano D. M. Oh W. (1972). Nursery outbreak of staphylococcal scalded skin syndrome: rapid identification of the epidemic bacterial strain. *Am. J. Dis. Child.* 124 41–44. 10.1001/archpedi.1972.02110130043006 5033748

[B4] Arndt D. Grant J. R. Marcu A. Sajed T. Pon A. Liang Y. (2016). PHASTER: a better, faster version of the PHAST phage search tool. *Nucleic Acids Res.* 44 W16–W21. 10.1093/nar/gkw387 27141966 PMC4987931

[B5] Azarian T. Cook R. L. Johnson J. A. Guzman N. McCarter Y. S. Gomez N. (2015). Whole-genome sequencing for outbreak investigations of methicillin-resistant *staphylococcus aureus* in the neonatal intensive care unit: time for routine practice? *Infect. Control Hosp. Epidemiol.* 36 777–785. 10.1017/ice.2015.73 25998499 PMC4507300

[B6] Azarian T. Maraqa N. F. Cook R. L. Johnson J. A. Bailey C. Wheeler S. (2016). Genomic epidemiology of methicillin-resistant *Staphylococcus aureus* in a neonatal intensive care unit. *PLoS One* 11:e0164397. 10.1371/journal.pone.0164397 27732618 PMC5061378

[B7] Botka T. Růžičková V. Konečná H. Pantůček R. Rychlík I. Zdráhal Z. (2015). Complete genome analysis of two new bacteriophages isolated from impetigo strains of *Staphylococcus aureus*. *Virus Genes* 51 122–131. 10.1007/s11262-015-1223-8 26135320

[B8] Botka T. Růžičková V. Svobodová K. Pantůček R. Petráš P. Čejková D. (2017). Two highly divergent lineages of exfoliative toxin B-encoding plasmids revealed in impetigo strains of *Staphylococcus aureus*. *Int. J. Med. Microbiol.* 307 291–296. 10.1016/j.ijmm.2017.05.005 28579477

[B9] Bradley P. den Bakker H. C. Rocha E. P. C. McVean G. Iqbal Z. (2019). Ultrafast search of all deposited bacterial and viral genomic data. *Nat. Biotechnol.* 37 152–159. 10.1038/s41587-018-0010-1 30718882 PMC6420049

[B10] Braunstein I. Wanat K. A. Abuabara K. McGowan K. L. Yan A. C. Treat J. R. (2014). Antibiotic sensitivity and resistance patterns in pediatric staphylococcal scalded skin syndrome. *Pediatr. Dermatol.* 31 305–308. 10.1111/pde.12195 24033633 PMC4349361

[B11] Cella E. David M. Z. Jubair M. Baines S. Pegues D. A. Azarian T. (2021). Complete genome sequence of an exfoliative toxin producing *Staphylococcus aureus* Strain MSSA_SSSS_01 from a case of staphylococcal scalded skin syndrome. *Microbiol. Resour. Announc.* 10:e01335-20. 10.1128/MRA.01335-20 33574109 PMC7883837

[B12] Chen C. Khaleel S. S. Huang H. Wu C. H. (2014). Software for pre-processing Illumina next-generation sequencing short read sequences. *Source Code Biol. Med.* 9:8. 10.1186/1751-0473-9-8 24955109 PMC4064128

[B13] Conceição T. Aires-De-Sousa M. Pona N. Brito M. J. Barradas C. Coelho R. (2011). High prevalence of ST121 in community-associated methicillin-susceptible *Staphylococcus aureus* lineages responsible for skin and soft tissue infections in Portuguese children. *Eur. J. Clin. Microbiol. Infect. Dis.* 30 293–297. 10.1007/s10096-010-1087-8 21046422

[B14] Croucher N. J. Page A. J. Connor T. R. Delaney A. J. Keane J. A. Bentley S. D. (2014). Rapid phylogenetic analysis of large samples of recombinant bacterial whole genome sequences using Gubbins. *Nucleic Acids Res.* 43:e15. 10.1093/nar/gku1196 25414349 PMC4330336

[B15] Dasenbrook E. C. Checkley W. Merlo C. A. Konstan M. W. Lechtzin N. Boyle M. P. (2010). Association between respiratory tract methicillin-resistant *Staphylococcus aureus* and survival in cystic fibrosis. *JAMA J. Am. Med. Assoc.* 303 2386–2393. 10.1001/jama.2010.791 20551409

[B16] Didelot X. Croucher N. J. Bentley S. D. Harris S. R. Wilson D. J. (2018). Bayesian inference of ancestral dates on bacterial phylogenetic trees. *Nucleic Acids Res.* 46:e134. 10.1093/nar/gky783 30184106 PMC6294524

[B17] Didelot X. Wilson D. J. (2015). ClonalFrameML: efficient inference of recombination in whole bacterial genomes. *PLoS Comput. Biol.* 11:e1004041. 10.1371/journal.pcbi.1004041 25675341 PMC4326465

[B18] Doudoulakakis A. Spiliopoulou I. Spyridis N. Giormezis N. Kopsidas J. Militsopoulou M. (2017). Emergence of a *Staphylococcus aureus* clone resistant to mupirocin and fusidic acid carrying exotoxin genes and causing mainly skin infections. *J. Clin. Microbiol.* 55 2529–2537. 10.1128/jcm.00406-17 28592549 PMC5527431

[B19] El Helali N. Carbonne A. Naas T. Kerneis S. Fresco O. Giovangrandi Y. (2005). Nosocomial outbreak of staphylococcal scalded skin syndrome in neonates: epidemiological investigation and control. *J. Hosp. Infect.* 61 130–138. 10.1016/j.jhin.2005.02.013 16009455

[B20] Florman A. L. Holzman R. S. (1980). Nosocomial scalded skin syndrome: Ritter’s Disease caused by phage Group 3 *Staphylococcus aureus*. *Am. J. Dis. Child.* 134 1043–1045. 10.1001/archpedi.1980.02130230023006 6449146

[B21] Goerke C. Pantucek R. Holtfreter S. Schulte B. Zink M. Grumann D. (2009). Diversity of prophages in dominant *Staphylococcus aureus* clonal lineages. *J. Bacteriol.* 191 3462–3468. 10.1128/JB.01804-08 19329640 PMC2681900

[B22] Gonzalez B. E. Rueda A. M. Shelburne S. A. Musher D. M. Hamill R. J. Hulten K. G. (2006). Community-associated strains of methicillin-resistant *Staphylococccus aureus* as the cause of healthcare-associated infection. *Infect. Control Hosp. Epidemiol. Off. J. Soc. Hosp. Epidemiol. Am.* 27 1051–1056. 10.1086/507923 17006811

[B23] Harkins C. P. Pettigrew K. A. Oravcová K. Gardner J. Hearn R. M. M. R. Rice D. (2018). The microevolution and epidemiology of *Staphylococcus aureus* colonization during atopic eczema disease flare. *J. Invest. Dermatol.* 138 336–343. 10.1016/j.jid.2017.09.023 28951239 PMC5780352

[B24] Harris S. R. Feil E. J. Holden M. T. G. Quail M. A. Nickerson E. K. Chantratita N. (2010). Evolution of MRSA during hospital transmission and intercontinental spread. *Science* 327 469–474. 10.1126/science.1182395 20093474 PMC2821690

[B25] Hultén K. G. Kok M. King K. E. Lamberth L. B. Kaplan S. L. (2019). Increasing numbers of staphylococcal scalded skin syndrome cases caused by ST121 in Houston. Texas. *Pediatr. Infect. Dis. J.* 39 30–34. 10.1097/inf.0000000000002499 31725120

[B26] Inouye M. Dashnow H. Raven L.-A. Schultz M. B. Pope B. J. Tomita T. (2014). SRST2: rapid genomic surveillance for public health and hospital microbiology labs. *Genome Med.* 6:90. 10.1186/s13073-014-0090-6 25422674 PMC4237778

[B27] International Working Group on the Classification of Staphylococcal Cassette Chromosome Elements (IWG-SCC) (2009). Classification of staphylococcal cassette chromosome mec (SCCmec): guidelines for reporting novel SCCmec elements. *Antimicrob. Agents Chemother.* 53 4961–4967. 10.1128/AAC.00579-09 19721075 PMC2786320

[B28] Jackson K. A. Gokhale R. H. Nadle J. Ray S. M. Dumyati G. Schaffner W. (2019). Public health importance of invasive methicillin-sensitive *Staphylococcus aureus* infections: surveillance in 8 US counties, 2016. *Clin. Infect. Dis.* 70 1021–1028. 10.1093/cid/ciz323 31245810 PMC7902232

[B29] Kanjilal S. Abdul Sater M. R. Thayer M. Lagoudas G. K. Kim S. Blainey P. C. (2018). Trends in antibiotic susceptibility in *Staphylococcus aureus* in Boston, Massachusetts, from 2000 to 2014. *J. Clin. Microbiol.* 56:e01160-17. 10.1128/JCM.01160-17 29093105 PMC5744217

[B30] Köser C. U. Holden M. T. G. Ellington M. J. Cartwright E. J. P. Brown N. M. Ogilvy-Stuart A. L. (2012). Rapid whole-genome sequencing for investigation of a neonatal MRSA outbreak. *N. Engl. J. Med.* 366 2267–2275. 10.1056/NEJMoa1109910 22693998 PMC3715836

[B31] Kuroda M. Ohta T. Uchiyama I. Baba T. Yuzawa H. Kobayashi I. (2001). Whole genome sequencing of meticillin-resistant *Staphylococcus aureus*. *Lancet* 357 1225–1240. 10.1016/S0140-6736(00)04403-2 11418146

[B32] Ladhani S. Joannou C. L. Lochrie D. P. Evans R. W. Poston S. M. (1999). Clinical, microbial, and biochemical aspects of the exfoliative toxins causing staphylococcal scalded-skin syndrome. *Clin. Microbiol. Rev.* 12 224–242. 10.1128/cmr.12.2.224 10194458 PMC88916

[B33] Malachowa N. Deleo F. R. (2010). Mobile genetic elements of *Staphylococcus aureus*. Cellular and molecular life sciences. *Cell Mo.l Life Sci.* 67 3057–3071. 10.1007/s00018-010-0389-4 20668911 PMC2929429

[B34] Mason A. Foster D. Bradley P. Golubchik T. Doumith M. Gordon N. C. (2018). Accuracy of different bioinformatics methods in detecting antibiotic resistance and virulence factors from *Staphylococcus aureus* whole-genome sequences. *J. Clin. Microbiol.* 56:e01815-17. 10.1128/JCM.01815-17 29925638 PMC6113501

[B35] Moradigaravand D. Jamrozy D. Mostowy R. Anderson A. Nickerson E. K. Thaipadungpanit J. (2017). Evolution of the *Staphylococcus argenteus* ST2250 clone in Northeastern Thailand is linked with the acquisition of livestock-associated staphylococcal genes. *mBio* 8:e00802-17. 10.1128/mBio.00802-17 28679748 PMC5573676

[B36] Murono K. Fujita K. Yoshioka H. (1988). Microbiologic characteristics of exfoliative toxin–producing *Staphylococcus aureus*. *Pediatr. Infect. Dis. J.* 7 313–315. 10.1097/00006454-198805000-00003 3380582

[B37] Nguyen L. T. Schmidt H. A. Von Haeseler A. Minh B. Q. (2015). IQ-TREE: a fast and effective stochastic algorithm for estimating maximum-likelihood phylogenies. *Mol. Biol. Evol.* 32 268–274. 10.1093/molbev/msu300 25371430 PMC4271533

[B38] Nübel U. Nachtnebel M. Falkenhorst G. Benzler J. Hecht J. Kube M. (2013). MRSA transmission on a neonatal intensive care unit: epidemiological and genome-based phylogenetic analyses. *PLoS One* 8:e54898. 10.1371/journal.pone.0054898 23382995 PMC3561456

[B39] Page A. J. Cummins C. A. Hunt M. Wong V. K. Reuter S. Holden M. T. G. (2015). Roary: rapid large-scale prokaryote pan genome analysis. *Bioinformatics* 31:btv421. 10.1093/bioinformatics/btv421 26198102 PMC4817141

[B40] Rao Q. Shang W. Hu X. Rao X. (2015). *Staphylococcus aureus* ST121: a globally disseminated hypervirulent clone. *J. Med. Microbiol.* 64 1462–1473. 10.1099/jmm.0.000185 26445995

[B41] Roisin S. Gaudin C. De Mendonça R. Bellon J. Van Vaerenbergh K. De Bruyne K. (2016). Pan-genome multilocus sequence typing and outbreak-specific reference-based single nucleotide polymorphism analysis to resolve two concurrent *Staphylococcus aureus* outbreaks in neonatal services. *Clin. Microbiol. Infect.* 22 520–526. 10.1016/j.cmi.2016.01.024 26899827

[B42] Růžičková V. Pantůček R. Petráš P. Machová I. Kostýlková K. Doškař J. (2012). Major clonal lineages in impetigo *Staphylococcus aureus* strains isolated in Czech and Slovak maternity hospitals. *Int. J. Med. Microbiol.* 302 237–241. 10.1016/j.ijmm.2012.04.001 22664376

[B43] Seemann T. (2014). Prokka: rapid prokaryotic genome annotation. *Bioinformatics* 30 2068–2069. 10.1093/bioinformatics/btu153 24642063

[B44] Sievers F. Higgins D. G. (2018). Clustal Omega for making accurate alignments of many protein sequences. *Protein Sci.* 27 135–145. 10.1002/pro.3290 28884485 PMC5734385

[B45] Staiman A. Hsu D. Y. Silverberg J. I. (2018). Epidemiology of staphylococcal scalded skin syndrome in U.S. children. *Br. J. Dermatol.* 178 704–708. 10.1111/bjd.16097 29077993

[B46] Tonkin-Hill G. Lees J. A. Bentley S. D. Frost S. D. W. Corander J. (2019). Fast hierarchical Bayesian analysis of population structure. *Nucleic Acids Res.* 47 5539–5549. 31076776 10.1093/nar/gkz361 PMC6582336

[B47] Wick R. R. Judd L. M. Gorrie C. L. Holt K. E. (2017). Unicycler: resolving bacterial genome assemblies from short and long sequencing reads. *PLoS Comput. Biol.* 13:e1005595. 10.1371/journal.pcbi.1005595 28594827 PMC5481147

[B48] Yamaguchi T. Hayashi T. Takami H. Nakasone K. Ohnishi M. Nakayama K. (2000). Phage conversion of exfoliative toxin A production in *Staphylococcus aureus*. *Mol. Microbiol.* 38 694–705. 10.1046/j.1365-2958.2000.02169.x 11115106

[B49] Yamaguchi T. Yokota Y. Terajima J. Hayashi T. Aepfelbacher M. Ohara M. (2002). Clonal association of *Staphylococcus aureus* causing bullous impetigo and the emergence of new methicillin-resistant clonal groups in Kansai District in Japan. *J. Infect. Dis.* 185 1511–1516. 10.1086/340212 11992289

[B50] Yoshizawa Y. Sakurada J. Sakurai S. Machida K. Kondo I. Masuda S. (2000). An Exfoliative toxin a-converting phage isolated from *Staphylococcus aureus* Strain ZM. *Microbiol. Immunol.* 44 189–191. 10.1111/j.1348-0421.2000.tb02481.x 10789506

