# Supplementary material for: Genomic Epidemiology and Global Population Structure of Exfoliative Toxin A-Producing Staphylococcus aureus Strains Associated With Staphylococcal Scalded Skin Syndrome
Source: Front Microbiol. 2021 Aug 18;12:663831. doi: 10.3389/fmicb.2021.663831 (PMC8416508; doi:10.3389/fmicb.2021.663831)
Supplement: Supplementary file 1 [file Data_Sheet_1.docx]

**Genomic epidemiology and global population structure of exfoliative toxin A (ETA) producing *Staphylococcus aureus* strains associated with Staphylococcal Scalded Skin Syndrome**

**Supplemental Table 1.** Table of sequence cluster (SC), the number of full-length prophage genomes (n=139) obtained from each SC, ΦETA group based on nucleotide identity clustering, and the closest matching published ΦETA genome.

| **Sequence Cluster** | **Classified phages** | **Phage groups** | **Related Phages** |
| --- | --- | --- | --- |
| SC1 | 4/5 | G14, G24 | phiETA3 (NC_008799) |
| SC2 | 15/72 | G02, G05, G06, G07, G13 | phiETA (NC_003288) |
| SC3 | 21/25 | G01, G11, G16, G20, G24, G26 | phiETA2 (NC_008798) |
| SC4 | 8/119 | G04, G09, G26, G27 | B166 (NC_028859) |
| SC5 | 11/20 | G15, G16 | phiETA2 (NC_008798) |
| SC6 | 0/6 | - | phiETA2 (NC_008798) |
| SC7 | 5/8 | G08 | *SA97 (NC_029010) |
| SC8 | 49/88 | G10, G19, G20, G22, G24, G25, G26 | phiETA3 (NC_008799), B166 (NC_028859) |
| Other | 15/34 | G11, G12, G18, G21, G23, G24, G26 |  |
| *Not an ETA carrying phage | | |  |

**Supplemental Figure 1.** Timeseries of case patient location, length of stay, and positive *S. aureus* culture in relation to healthcare worker contact. Shading represents the length of stay, scaled in weeks, and is shaded based on three separate bays within the neonatal intensive care unit. Diamonds represent contact with the colonized healthcare worker and microscope symbol denotes the week of the positive laboratory result.

**Supplemental Figure 2. Core genome phylogeny, region of isolation, and phage phylogroup for major sequence clusters (SCs).** For each major SC, a core genome maximum likelihood phylogeny was inferred. The number of core genes, length of core genome alignment, and single nucleotide polymorphism (SNP) diversity is annotated on each phylogeny. The outbreak strains are annotated on the SC8 phylogeny illustrating ST582. The heatmap shows the region of isolation (inside bar) and the ΦETA phylogroup. Note that isolates from the United Kingdom (UK) are indicated separately from the rest of Europe. Multilocus sequence type (MLST) is annotated on the tree.

**Supplemental Figure 3. Core genome phylogeny, region of isolation, and phage phylogroup for minor sequence clusters (SCs).** For each minor SC, a core genome maximum likelihood phylogeny was inferred. The number of core genes, length of core genome alignment, and single nucleotide polymorphism diversity (SNP) diversity is annotated on each phylogeny. The heatmap shows the region of isolation (inside bar) and the ΦETA phylogroup. Note that isolates from the United Kingdom (UK) are indicated separately from the rest of Europe. Multilocus sequence type (MLST) is annotated on the tree.

**Supplemental Figure 4.** Root-to-tip analysis of *eta*-positive ST582 strains (n=30) for temporal signal. The left panel shows the maximum likelihood phylogeny inferred from a recombination free alignment. Tip points are shaded as a color ramp representing date of collection with older (blue), purple (intermediate) and more recent (red). The green tip color represents a strain with an unknown date of collection. The right panel shows the regression of root-to-tip tree distance (SNPs) on the year of collection with the tips colored as in the panel on the left. The sample showed significant temporal signal (Pearsons correlation coefficient, R^2^ = 0.70, p<0.0001). The estimated mutation rate is 4.39 SNPs/genome/year with a date of the most recent common ancestor of 1993.7.

**Supplemental Figure 5. Tanglegram comparing the core genome phylogeny and ΦETA gene tree.** Dendroscope was used to generate a tanglegram of the core genome phylogeny shown in Figure 2A to the ΦETA phage gene tree in Figure 3A. Dominant lineages are colored accordingly and are annotated with MLST and SC. Lines illustrate differences in phage content across the *S. aureus* population.
